# Current Efficacy of Multiepitope Vaccines Against Helminths: A Systematic Review

**DOI:** 10.3390/biom15060867

**Published:** 2025-06-13

**Authors:** Ignacio Trujillo-Rodríguez, Julio López-Abán, Montserrat Alonso-Sardón, Belén Vicente-Santiago, Antonio Muro-Álvarez, Raúl Manzano-Román

**Affiliations:** 1 Infectious and Tropical Diseases Research Group (e-INTRO), Biomedical Research Institute of Salamanca Research Centre for Tropical Diseases at the University of Salamanca (IBSAL-CIETUS), 37007 Salamanca, Spain; igntrurod@usal.es (I.T.-R.); belvi25@usal.es (B.V.-S.); ama@usal.es (A.M.-Á.); 2 Área de Medicina Preventiva, Epidemiología y Salud Pública, Facultad de Medicina, CIETUS, e-INTRO, IBSAL, Universidad de Salamanca, 37007 Salamanca, Spain; sardonm@usal.es

**Keywords:** helminths, vaccines, multiepitope, in vivo efficacy, systematic review

## Abstract

**Background**: Vaccination represents an efficient way to control communicable diseases. Reliable vaccines would reduce the use of anthelmintics drugs and fight against the concern of anthelmintics resistances. Unfortunately, anthelmintic vaccines face many difficulties in their development. One of the most innovative vaccine models in this field is multiepitope vaccines since, based on advances in immunoinformatics, they facilitate immunization against parasites at different stages of their cycles. **Objective**: In this study, we evaluate the published efficacy of multiepitope vaccines against helminths. **Methods**: Independent reviewers conducted a comprehensive search of multiple databases until September 20th 2024, following PRISMA 2020 guidelines. The review included original in vivo protection studies using chimeric vaccines with antigenic epitopes in experimental models. Key information was summarized, tabulated, and analyzed, and risk of bias was assessed using the SYRCLE risk tool. **Results**: A total of 15 preclinical studies were included. In those immunization experiments, parasite load reductions varied from 12.4% to 100%. **Conclusions**: Overall, this study shows protections in parasite load or lesion in 50–80% and significant survival rates using experimental vaccines including B- and T-cell epitopes in a wide range of helminthic infections. Given the variability of the experiments and the limited available data, there was not a clear correlation between protections and immune responses. Confirmation trials are needed to corroborate the protection and immunological mechanisms reached not only in this initial valuable study but also with other multiepitope candidates.

## 1. Introduction

Since the dawn of humanity, helminth parasites have accompanied us, impacting both our health and that of our domestic animals [1]. Currently, millions of people live in high-risk areas of infection, and animal production in grazing areas is compromised by helminth infections. The current treatment and control measures rely on anthelmintic drugs application, based on benzimidazoles, macrocyclic lactones, depsipeptides, imidazothiazole derivatives, and amino-acetonitrile derivatives, among others. However, effectiveness in human populations is limited due to the difficulties in reaching a high coverage. In livestock, reinfections have high frequency, and there is the issue of the recurrent emergence of drug-resistant strains [2]. Historically, vaccines have proven to be highly effective against viral and bacterial diseases, serving as a preventive, safe, and efficient method for controlling epidemics and animal production losses [3]. Unfortunately, vaccines against helminths have been in development for a long time with poor results.

The traditional pipeline based on attenuated strains or purified antigens has yielded one licensed vaccine against *Dictyocaulus viviparus* using attenuated infective larvae, another against *Haemonchus contortus* using native gut antigens, and another against *Echinococcus granulosus* using the EG95 protein [4]. In the last few decades, based on massive genomic and proteomic data, a hypothesis-driven design for vaccines was developed [5]. This approach is based on the in silico selection and evaluation of candidate vaccine epitopes. This vaccine technology involves the bioinformatic design of antigenic chimeras, composed of various antigenic epitopes assembled into multiepitope vaccines (MEVs). Also, these vaccines have been produced as recombinant, as nucleic acids, chemically synthesized, or included in bacterial plasmids and then assessed for their safety and immunological capabilities. Multiepitope vaccines are those that include sequences of peptides putative to develop immunological responses through B- and T-cell mechanisms. Within the host organism, the polypeptide is processed to induce a potential protective immune response after interaction with several MHC haplotypes [6]. This technology allows targeting organisms with high complexity and variability in their life cycles, overcoming the problems related to the use of live or attenuated parasites [7]. Helminths exhibit a wide variety of antigens at different stages of their life cycle, making them ideal candidates for the development of MEVs [8]. Researchers worldwide have focused their efforts on identifying antigenic combinations that elicit consistent and effective immune responses against various helminths. Infectious challenges are carried out to detect a significant effect on parasite burden, lesion, or immunological response, either humoral or cellular.

This systematic review presents the advancements in multiepitope vaccines against trematode, cestode, and nematode experimental infections. Protection in terms of parasite burden and humoral and cellular immune response were assessed.

## 2. Materials and Methods

### 2.1. Protocol and Registry

A search was carried out to identify whether a similar study existed using the International Prospective Register of Systematic Reviews database. Since no similar article was found, we proceeded to register this review (PROSPERO: CRD420251001796). This systematic review was conducted in accordance with the PRISMA 2020 guidelines (Preferred Reporting Items for Systematic Reviews and Meta-Analyses) [9] and the recommendations of the Cochrane Handbook for systematic reviews [10].

### 2.2. Research Question

Our study deals with the efficacy of multiepitope vaccines developed to protect against trematodes, cestodes, and nematodes in experimental models. Multiepitope vaccines are considered those that include sequences of peptides putative to develop immunological responses through B- and T-cell mechanisms affecting antigen presentation. This review includes antigen designs, formulations, and trials with experimental models useful for detecting protection with the minimum number of animals in each group. Challenges were carried out to detect a significant effect on parasite burden, lesion, or immunological response, either humoral or cellular.

The research question was defined using the PICO structure: study Population (P): experimental models; Intervention (I): vaccination with multiepitope vaccines formulated in different adjuvant systems and challenged with a parasite inoculum; Outcomes (O): variables of parasite burden, lesions, and immunological response to measure vaccine efficacy; and Compare (C): with animals treated with the adjuvant system and challenged comparably to the intervention but without antigens. Thus, the guiding question of this study was “Do multiepitope vaccine trials in animal experimental models of helminthiasis provide evidence of protective, secure, and correlated immune responses?”.

### 2.3. Eligibility Criteria

We meticulously defined eligibility criteria rooted in the PICO framework to ensure a comprehensive and targeted analysis. Inclusion criteria: We included studies focused on development of a vaccine against pathogenic helminth for humans, assessed vaccines composed with different antigenic epitopes, and carried out in vivo protection and safety studies. Exclusion criteria: Studies were excluded that did not really answer our question or were not really multiepitope vaccines; reports such as meeting abstracts, reviews, letters, dissertation theses, editorial materials, books, or news; and research that only had in vivo immune response studies, in silico studies, or in vitro studies.

Significant restrictions on study eligibility were carefully justified. The search strategy was exhaustive and did not involve querying study registries, regulatory databases, or additional online repositories with data restrictions. No organizations or individuals were contacted to identify studies for inclusion. In addition, reference lists of relevant study reports and systematic reviews on similar topics were reviewed to ensure completeness.

### 2.4. Information Sources and Search Strategy

The last search was conducted on September 20th 2024. A search strategy was detailed in the PubMed (MEDLINE) and Web of Science browsers (Web of Science Core Collection, MEDLINE, Current Contents Connect, SciELO Citation Index, Grants Index). Search terms covered title, abstract, and keywords. A variety of topic keywords and Boolean operators (AND, OR) were used in the Topic field: “vaccin*” AND (“multiepitop*” OR “multi-epitop*” OR “multivalent*” OR “multi-valent*”) AND (“fasciol*” OR “schistosom*” OR “clonorch*” OR “paragonim*” OR “heteroph*” OR “opisthorch*” OR “metagonim*” OR “fasciolop*” OR “echinococ*” OR “hydatid*” OR “taen*” OR “cysticerc*” OR “diphyllobot*” OR “enterobi*” OR “pinwor*” OR “brugi*” OR “filari*” OR “ascari*” OR “trichine*” OR “haemonch*” OR “wuchereri*” OR “trichuri*” OR “whipwor*” OR “ancylostom*” OR “hookwor*” OR “necato*” OR “hookwor*” OR “strongyloid*” OR “anisaki*” OR “dracuncul*”). There were no publication date limits or language restrictions. Details of the strategy and search equation used are available (Table S1). Web of Science automation tools were used to exclude meeting abstracts, letters, doctoral theses, editorial material, books, and news.

### 2.5. Selection Process of the Studies

Two independent reviewers screened each record (J.L-A. and I.T-R.), ensuring a robust selection process. Disagreements were resolved through joint discussion. Primarily, we eliminated duplicate records obtained from the databases. The remaining records were screened by title and abstract, and irrelevant records were eliminated. Following the eligibility and inclusion criteria, eligible records were selected for full-text download.

### 2.6. Data Extraction

Data were extracted from the papers and collected using a Microsoft Excel worksheet, and duplicates were discarded. Data were meticulously compiled into a list of key variables for the analysis: parasite, vaccine type, animal model and size, country, authors and year of publication, antigen, adjuvant system, administration dose, route and schedule of vaccination, challenge, vaccine efficacy, and humoral and cellular response.

### 2.7. Risk of Bias

The SYRCLE-RoB-tool (Systematic Review Center for Laboratory Animal Experimentation Risk of Bias tool) was used to assess the risk of bias in laboratory animal studies with randomized or observational designs [11]. This tool considers ten entries grouped into six domains or types of bias (some of them include more than one entry in the same domain): selection bias (1, 2, and 3), performance bias (4 and 5), detection bias (6 and 7), attrition bias (8), reporting bias (9), and other sources of bias not covered by other domains in the tool (10). A “yes” answer indicates a low risk of bias; a “no” answer indicates a high risk of bias; the “unclear” answer indicates that insufficient detail has been reported to adequately assess the risk of bias. By assessing these ten entries, the SYRCLE-RoB tool provides a framework for assessing the risk of bias in animal intervention studies, allowing researchers to better interpret the validity of the evidence. This step was also blindly and independently performed by two reviewers (J.L-A. and I.T-R.), and divergent results were resolved by consensus.

### 2.8. Data Synthesis and Analysis

First, a structured conceptual synthesis of the data extracted from each selected article was performed, followed by comparisons between studies based on the data extraction table. Meta-analysis and heterogeneity analyses were not performed. We made every effort to confirm the relevance and accuracy of the data extracted, ensuring that the conclusions of this systematic review were well-founded and reliable.

## 3. Results

### 3.1. Study Selection

**Figure 1:**
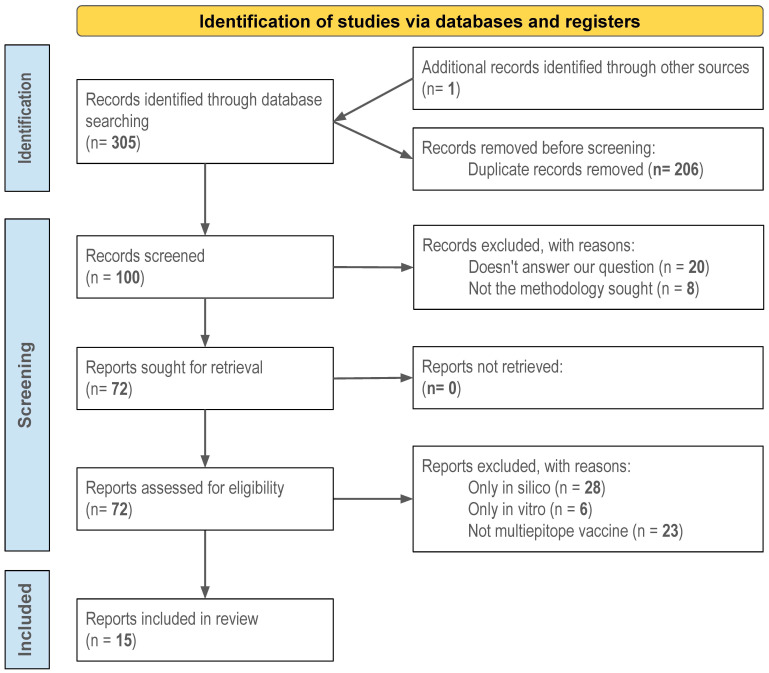
PRISMA flow chart of the study selection and inclusion process.

The preliminary search identified 306 papers in PubMed and Web of Science. After removing 206 duplicate articles, 100 potentially relevant papers remained. After screening the title and abstract, 28 records were excluded for the following reasons: 20 did not answer our question, and 8 did not follow the methodology sought. Of the 72 articles screened for eligibility, 23 articles were excluded due their lack of focus on multiepitope vaccines, 28 for conducting in silico analysis, and 6 for only performing in vitro evaluations. Finally, 15 articles with 23 studies were included for qualitative assessment (Figure 1).

### 3.2. Study Characteristics

**Figure 2:**
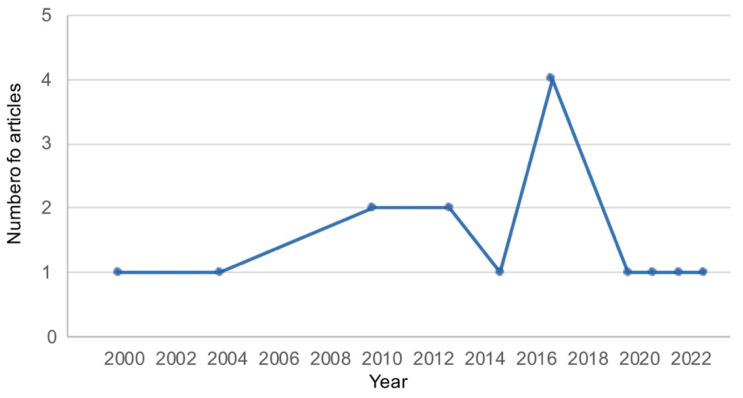
Number of articles on multiepitope vaccines carried out against helminths: in vivo models and date of publication.

The main characteristics of the 15 included articles are summarized in Table 1. However, we needed to split some of them to analyze the different studies they carried out, making a total of 23 studies. The first selected article was published in 2000. The rest of the studies were conducted since then, although the number of publications increased from 2010 onwards (Figure 2).

The parasites studied were varied, comprising mostly *Brugia malayi* with four studies [12,13,14,15], *Fasciola hepatica* with another four studies [16] and *Trichinella spiralis* with four studies as well [17,18,19]. The genus *Echinococcus* is represented in four studies, with three of the species being *Echinococcus granulosus* [20,21] and one *E. multilocularis* [22]. Two studies deal with *Taenia solium* [23], one with *T. crassiceps* [23], and another three with the genus *Schistosoma*, with two on *S. mansoni* [24] and one on *S. japonicum* [25]. Finally, one study was found on *Ascaris suum* [26].

**Table 1:** Parasites (*Ascaris suum*, *Brugia malayi*, *Echinococcus granulosus*, *E. multilocularis*, *Fasciola hepatica*, *Schistosoma japonicum*, *S. mansoni*, *Taenia solium*, *T. crassiceps*, and *Trichinella spiralis*), vaccine design, animal model and size, country, and authors of the studies included.

Parasite	Vaccine Type	Animal Model	Nº Animals (nº per Group)	Country	Authors, Publication Year [Reference]
Trematodes					
*F. hepatica*	Mixture synthetic peptides	Mouse, CD1	49 (7)	Spain	Rojas-Caraballo et al., 2017 [16]
*F. hepatica*	Mixture synthetic peptides	Mouse, CD1	49 (7)	Spain	Rojas-Caraballo et al., 2017 [16]
*F. hepatica*	Mixture synthetic peptides	Mouse, CD1	49 (7)	Spain	Rojas-Caraballo et al., 2017 [16]
*F. hepatica*	Mixture synthetic peptides	Mouse, CD1	49 (7)	Spain	Rojas-Caraballo et al., 2017 [16]
*S. japonicum*	Plasmid pET32a	Mouse, BALB/c	55 (11)	China	Guo et al., 2010 [25]
*S. mansoni*	Synthetic peptide epitope-based polymers	Mice, CBA, BALB/c	72 (12)	Australia	Yang et al., 2000 [24]
*S. mansoni*	DNA vaccine encoding different epitopes in tandem	Mice CBA, BALB/c, C57BL/6J	48 (8) or 61 (10)	Australia	Yang et al., 2000 [24]
Cestodes					
*E. granulosus*	Recombinant multiepitope (rEGVac)	Dog	15 (3)	Iran	Pourseif et al., 2021 [20]
*E. granulosus*	Recombinant multiepitope (rEGVac)	Sheep	15 (3)	Iran	Pourseif et al., 2021 [20]
*E. granulosus*	Recombinant fusion polypeptide (ChMEA)	Mouse, BALB/C	20 (5)	Iran	Esmaelizad et al., 2013 [21]
*E. multilocularis*	Recombinant multiepitope (rMEV) (GILE)	Mice, SWISS, BALB/c	12 (6)	China	Zhou et al., 2023 [22]
*T. crassiceps*	DNA fragments in phage vector (CPhV)	Mouse, BALB/cAnN	18 (6)	Mexico	Manoutcharian et al., 2004 [23]
*T. solium*	DNA fragments in phage vector (CPhV)	Pig	12 (3)	Mexico	Manoutcharian et al., 2004 [23]
*T. solium*	DNA fragments in phage vector (CPhV)	Pig	12 (3)	Mexico	Manoutcharian et al., 2004 [23]
Nematodes					
*A. suum*	Recombinant multipeptide (ASCVac-1)	Mouse, BALB/c	64 (16)	Brazil	Gazzinelli-Guimaraes et al., 2022 [26]
*B. malayi*	Chimeric epitope gene construct (FEP)	Mongolian jirds	10 (5)	India	Anugraha et al., 2015 [12]
*B. malayi*	Recombinant multiepitope (rAEP)	*Mastomys coucha*	18 (6)	India	Madhumathi et al., 2017 [13]
*B. malayi*	Conjugated synthetic peptides (PC1)	*Mastomys coucha*	18 (6)	India	Madhumathi et al., 2010 [14]
*B. malayi*	Synthetic multi-antigen peptide (TT MAP)	Mongolian jird	25 (5)	India	Immanuel et al., 2017 [15]
*T. spiralis*	Multiple antigen peptide (MAP-TB)	Mouse, BALB/c	30 (10)	China	Gu et al., 2020 [17]
*T. spiralis*	Multiple antigen peptide (MAP-B)	Mouse, BALB/c	30 (10)	China	Gu et al., 2020 [17]
*T. spiralis*	KLH conjugated peptides	Mouse, BALB/c	48 (6)	China	Gu et al., 2013 [18]
*T. spiralis*	Recombinant multiepitope (rMEV)	Mouse, BALB/c	36 (12)	China	Gu et al., 2017 [19]

Most vaccines were based on synthetic peptides, numbering 10 studies [14,15,16,17,18,24], followed by recombinants, numbering 7 [13,19,20,21,22,26]. One study was performed on a DNA vaccine encoding different epitopes in tandem [24], three on DNA fragments in phage vector [23], one using a plasmid construct [25], and one study with a chimeric vaccine [12].

Different experimental models were used prior to the final clinical trials for the evaluation of the effectiveness and safety of the formulated antigens. The predominant animal model was mice, representing 17 studies, of which 11 used the syngeneic BALB/c [17,18,19,21,22,23,24,25,26], CBA (2 studies) [24], and C57BL/6J (1 study) mouse strains [24]. Two studies used outbreed mice strains: four with CD1 [16] and one with SWISS [22]. Other models were pigs (two studies) [23], Mongolian jirds (two studies) [12,15], *Mastomys coucha* (two studies) [13,14], dogs (one study) [20], and sheep (one study) [20].

The number of individuals in the studies varied in each group, ranging from 3 [20,23] to 16 [26] animals per group. The majority of studies had six animals per group (in five studies) [13,14,18,22,23].

Among countries, China contributed the most with five papers [17,18,19,22,25], followed by India with four papers [12,13,14,15], and Iran with two papers [20,21]. Australia contributed one paper [24], Brazil contributed one paper [26], Spain contributed one paper [16], and Mexico contributed one paper [23].

### 3.3. Risk of Bias Assessment

**Figure 3:**
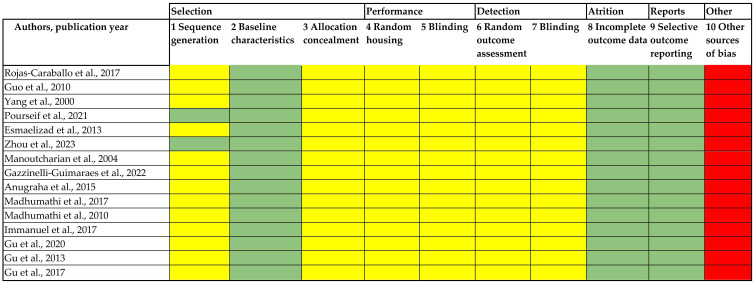
Risk of bias assessment of the included studies using the SYRCLE-RoB standard [12,13,14,15,16,17,18,19,20,21,22,23,24,25,26]. Green (low risk of bias), yellow (uncertain risk of bias), and red (high risk of bias).

The “performance” and “detection” of risk bias assessment domains indicate an uncertain risk due to insufficient information or inadequate descriptions in the studies evaluated. In contrast, all studies showed a low risk of bias in the areas of “selection”, “attrition”, and “reports” bias domains. In the “selection” domain, all studies provided sufficient information (low risk of bias) on the baseline characteristics of the animal models, and only two studies [20,22] provided detailed information on sequence generation (Figure 3). No studies were disqualified due to high risk of bias.

### 3.4. Synthesis of Results

The details of the main results of the 23 studies out of the 15 articles included in the systematic review are presented in Table 2. Most of the included studies, i.e., 12 studies, employed both T and B epitopes [12,14,15,16,17,19,20,22,24]. Four used B epitopes [13,16,17,26], two used T epitopes [16,21], and five did not clearly describe their origin [18,23,25]. Most studies used antigens with homology between species of the same genus, except for one study that reported the specificity of its epitopes for *T. crassiceps* also serving for *T. solium* [23].

The adjuvant system most used was Freund’s adjuvant, employed in seven studies [17,20,21,22,24]. Four studies used alum salts [12,13,14,15], while two studies used Montanide ISA 50 V2 SEPPIC [18,19] and one Montanide ISA 206 [25] oleous adjuvants. Four studies used the adjuvant adaptation (ADAD) adjuvant [16], and one study used an adjuvant based on *B. pertussis* monophosphoryl lipid A (BpMPLA) [26]. Four studies did not use any adjuvant system [23,24].

For vaccine administration, the most commonly chosen route was subcutaneous (s.c.), with 15 studies [16,17,18,19,20,21,23,25,26], followed by 5 studies using the intraperitoneal (i.p.) route [12,13,14,15,22], 2 studies using the intramuscular (i.m.) injection [24], and 1 study using the oral (v.o.) application [23]. In dosing, 12 studies stablished three doses [16,17,18,19,21,24,25], 6 studies four doses [12,13,14,15,22,23], and 5 studies applied only two doses of the vaccine [20,23,26]. In vaccination-booster intervals, most studies used a 2-week interval, with 14 studies [12,16,17,18,19,21,23,24,25], followed by 4 studies with a 1-week interval [13,14,15,22], 2 studies with 4-week intervals [20], and 1 study with 3-week intervals [24]. Also, there were two studies in which intervals were outside the weekly norm, with a schedule with applications on 0, 12, 23, and 34 days [23] and another one at 10 days [26].

Challenges were performed with embryonated eggs via oral (v.o.) in *A. suum* [26]; with infective third-stage larvae of *B. malayi* implanted in a microscopic chamber in the peritoneum [12,13,14,15]; with protoscoleces implanted intraperitoneally or eggs orally administered of *E. granulosus* or *E. multilocularis* [20,21,22] or metacercariae v.o. of *F. hepatica* [16]; with cercariae of *S. mansoni* or *S. japonicum* administered percutaneously or injected [24,25]; with cysticerci implanted in the peritoneum cavity of *T. crassiceps* [23]; with eggs v.o. in *T. solium* [23]; or with first-stage muscular larvae v.o. in *T. spiralis* [17,18,19].

Efficacy was recorded in almost all the studies in terms of reductions in the number of recovered parasite forms, but the weight of parasite burden, hepatic lesions, or survival of infected animals were used additionally. Only four studies showed very high efficacy in terms of parasite burden (>80%) [20,21,23]. Most vaccines showed high efficacy (50–80%) in nine studies [12,13,14,15,16,19,22,23,25]. Eight studies showed low efficacy (<50%) [16,17,18,23,26], and two studies showed no protection [24].

The humoral immune response indicates there high IgG responses against the multiepitope antigens in 11 studies [12,16,17,18,19,20,22,23,24,25]. When IgG subtypes were studied, we found nine studies reporting an increase in specific IgG1 [12,13,15,16,17,18,19,26], and one study reported an increase in specific IgG3 [26]. Moreover, we found one study showing high levels of specific IgG2 [19], two studies with specific IgG2a [12,17], and two studies with specific IgG2b [12,13]. There were also three studies reporting an increase in specific IgE [20,26] and one study with increase in IgM [15].

Cellular immunological response was determined in 16 out of the 23 recorded studies. In all of them, cytokines such as interleukin (IL)-2, IL-4, IL-5, IL-6, IL-8, IL-10, IL-12, IL-13, and IFN-γ were studied. We observe that there was an increase in IFN-γ in seven studies [12,15,17,19,22,23], IL-2 in three studies [14,15,17], IL-4 in eight cases [15,17,19,20,23,26], IL-5 in six studies [12,14,15,17,19,26], IL-6 in one study [17], IL-8 in one study [16], IL-10 in one study [15,16], IL-12 in one study [16], and IL-13 in one report [26]. Regarding the study of immune cells, only five reports where on lymphocyte and splenocyte proliferation; circulant lymphocytes, eosinophils, or neutrophils and Tfh, GC, Tfr, and Treg cells were also assessed. The data showed that vaccinated and challenged experimental animals had increased Tfh cells in two studies [17], GC cells in two studies [17], CD4+ cells in one study [22], and CD8+ cells in one study [22]. Lymphoproliferation was reported in three studies [23,26], increased splenocytes were found in two studies [12,15] and eosinophils reported in one study [26].

## 4. Discussion

Alternative control strategies based on protective immune responses upon immunization with parasite antigens is a realistic goal in view of the important roles many parasitic genes play in processes like development, parasitism, and reproduction. However, only an advanced vaccine called R21 exists in humans against plasmodium malaria, reaching around 68–75% efficacy in clinical trials, although a number of challenges exist for long-term success in high-risk areas, including on-time booster doses applications [27]. Therefore, the difficulties in achieving protection against complex metazoans like helminths are much greater. Mostly, because helminths have complex life cycles (involving adult and larval stages occupying different tissues in their definitive or intermediate host) and strategies to ensure survival, many of them are redundant and related to immune evasion and metabolic reprograming [28,29,30].

Classical, empirical trial-and-error approaches have guided the advancements with vaccine candidates based on single or a few different recombinant antigens, mainly for livestock in the attempt to reduce morbidity [4,31]. In this sense, it seems that targeting many antigens is necessary taking into account (i) the high biological complexity of helminths, (ii) the inherent limitations in obtaining native antigens, (ii) the fact that parasites release hundreds of diverse E/S products and molecules, (iii) the possibility that some of the E/S products are associated with the inhibition of protective responses [32,33], and (iv) the recognition that early initiation of a solid and broad immune response facilitates protection; all these may basically explain why the development of effective vaccines has been so difficult [34,35,36].

Previous facts have stimulated novel strategies for immunization to induce mixed Th1/Th2 immune response following experimental challenges using detailed omics metadata and potent bioinformatics tools to first identify potential vaccine candidates in helminths, theoretically making possible prophylactic, anti-pathology, and transmission blocking efficacies within a unique combinatorial vaccine [36,37]. All these allow searching specific sequences, comparing genomes in the same or related parasitic species, and predicting on a large scale the basic functional and targeted key molecules and determinants potentially inducing protective immunity [38,39]. However, despite intensive efforts to identify candidate epitopes using reverse vaccinology and to design multiepitope constructs, the immense majority of the publications only present theoretical MEVs without in vivo validations (Figure 1), thus hampering their real vaccinal value upon delivery with different platforms. Moreover, each has important processing challenges, like the case with mRNA vaccines [40].

In this study, we collected the original research on in vivo-validated multiepitope vaccines (MEVs) developed against helminths. We offer insights on efficacy against experimental infection and immunological responses of ten helminth species, three of them affecting man (*S. mansoni*, *S. japonicum*, and *B. malayi*), six zoonotic (*E. granulosus*, *E. multilocularis*, *T. solium*, *T. spiralis*, *T. crassiceps,* and *F. hepatica*), and another species affecting swine (*A. suum*). Most of the multiepitope candidates presented in this study are synthetic peptides elaborated in chimeric construct or administered as a mixture of them, but there are also recombinant proteins, plasmids, or phage constructs probably because of they are easier to obtain, manage in a laboratory, and formulate [41]. Protective responses induced by parasite immunomodulators are the foundation for advancement in vaccine development [8]. Both conjugated or mixtures of synthetic potentially immunogenic peptides (B- and T-cell epitopes) coming from E/S essential and immunomodulatory products with a previous history of protection trials as vaccines have provided partial protection administered with different adjuvants, as in the cases against *F. hepatica*, *B. malyi*, and *T. spiralis* (Table 1 and Table 2). Suitable adjuvants—we observed that mineral oils and squalene with amphiphilic molecules act as emulsifiers—induce the production of high antibody titers in the majority of these trials, making them useful to monitor immunogenicity but not enough to understand the mechanisms of protection, especially if there is no rigorous testing with standardization in protocols and assays [35,42]. Even so, parasite challenges at high doses may exert an important immune suppression effect, potentially impacting specific immune responses induced by vaccination, which emphasizes the importance of selecting appropriate models reflecting natural host conditions. And the optimal design of potentially broadly protective vaccines necessitates detailed knowledge of vaccine-induced immunity, which is not a general rule. Immunity to helminths requires both enhanced innate and adaptive mechanisms to achieve effective anti-helminthic vaccines, which has been called trained immunity [29].

In the case of the use of recombinant MEVs, it is worth mentioning the trial with a polypeptide comprising B- and T-cell epitopes of key *E. granulosus* proteins, which is partially protective. This polypeptide administered with Freund’s adjuvant provided an interesting 100% reduction in adult cestodes in dogs and 85% reduction in cysts in sheep, with increases in humoral antibodies (IgG/IgE and IL-4). It seems that IL-4 and humoral response to worms do not correlate, and increased antibody levels are a general and consistent response in almost all helminthic vaccine candidates [43]. This was also observed in the selected MEVs and could be correlated with worm burden and partial protection due to mixed Th1/Th2 responses, albeit isotype levels do not allow crucial distinction of the type of the immune responses. Antibody avidity is potentially an important analysis for achieving correlation of parasitism and reproduction with vaccine efficacy [33,44,45]. In this sense, it appears important in future research to differentiate between cross-reactive antigenicity and cross-protective immunogenicity regarding peptide targeting and to develop neutralization assays by polyclonal antipeptide antibodies targeting conformationally disordered B-cell epitopes [46,47]. IgE and IL-4 initially do not serve as correlates of protection because they are characteristic of Th2 immune responses and are not protective upon infection by parasitic worm helminths. However, further assays could define if IgE off-target effects against parasite determinants acting as allergens serve as correlates of protection [48]. In the case with a similar vaccine against *E. multilocularis*, variations in cellular immune markers also support the need for in-depth studies of the immune responses to identify real correlates of enhanced immunity from both innate and adaptative responses, facilitating early-stage targeting to further avoid parasite-induced type 2 [49].

DNA vaccination is another interesting and controllable platform for improving vaccine efficacy. Here, an important point is that, opposed to the case of protozoan parasites, so few DNA-based MEVs have been tested against helminth parasites in vivo, and the test mostly occurred several decades ago. Very relevant are the pioneering results with the bacteriophage vaccine developed against *T. solium* that reached 95% muscle cysticerci reductions in pigs without the use of adjuvants. This could be the cause of the observed immune response that was not fully in accordance with more recent correlates of cysticercosis protection, including low levels of IgG together with high levels of IL-4 [50]. However, this is a preliminary test with an interesting expression system that displays peptides in the surface of its particles, serving as a potent delivery system that in turn needs further validations with parasite MEVs [51].

Considering all of the above-mentioned research, an important limitation is the lack of in-depth immune response data to obtain clear correlates of vaccine immunogenicity and efficacy that might translate to the real hosts. Cellular immune response was studied with nine cytokines in nine cell populations. Cytokines and cells related to Th1, Th2, and Treg responses have not been systematically studied [52,53]. And correlates of immunity based on Th1/Th2 balanced responses in these high-dose challenges may not be informative about their role in immunity and tolerance [52]. This highlights the need to include in the protection trials robust immune-monitoring studies covering the specific induced immunological responses upon vaccination and infection to assess durable cell immunity and allow precision immune engineering using multiepitope vaccines in experimental models [54,55]. Although efficacy measured in terms of reduction in the parasitic load, lesions, and survival is widely used and directly related to the highly valuable protection level, the obtained knowledge must be considered preliminary and needs repetition of the best results and proof-of-concept trials with a higher number of animals and well-designated immunological assays [6,56].

## 5. Conclusions

Despite the limitations observed and exposed throughout the discussion of the included research—methodological in nature due to an insufficient sample size or heterogeneity of the vaccination protocols and in the results where antigenic variability, the use of clinically inapplicable adjuvants, or immunological evasion of the parasites compromise the efficacy of the vaccines—the studies reviewed show that MEVs are a promising alternative for the prophylaxis and control of helminth-caused diseases, as they offer the possibility of inducing a more robust and specific immune response against these parasites, making the vaccines more effective in stopping the spread and preventing transmission to the community.

A valuable number of MEV candidates reaching an effectiveness around 50–80% in terms of recovered parasite burden supports the possibility to counteract the parasites’ biological complexity and promissory advances in this field. Even then, researchers have to continue looking for new hidden or protected antigens with immunogenic peptides with important roles at the host–parasite interface. Exosomes provide a theoretical basics for the prevention and treatment of infectious diseases via MEVs [57]. In the case of complex organisms like helminths, the inclusion of modern immunopeptidomics approaches for specific immunogenicity validation of peptide candidates and nanostructured delivery systems is improving to induce fast, broad, and convenient immune protective responses [36,58].

Altogether, these approaches also highlight the need to follow well-suited and advanced protocols and a combination of techniques for the selection of comprehensive epitopes and for the design of multiepitope-based subunit vaccines as well as validations through other techniques to avoid some of the disadvantages of bioinformatic tools, like overprediction of epitopes [59,60]. Finally, further validations of the in silico-designed vaccines are essential to provide transferable anti-helminthic vaccines. In this sense, it is crucial to train and integrate bioinformatic algorithms well for future predictions involving complex parasites and to perform in vivo validation for plausible recombinant polypeptides with different adjuvants, especially modern self-adjuvating molecules like nanoparticles and liposomes, for the development of potentially long-lasting protective nanovaccines [61].

On the other hand, confirmation through ample-sized trials and sound, long-term immunological studies including Th17 or Treg responses for better in-depth understanding of the drivers and correlates of immunity is needed to validate MEV results before proof-of-concept trials under natural conditions can be carried out [43]. As mentioned, a number of important variables must be considered along with uniform criteria among research laboratories to make possible comparisons. The publication number and the chronological analysis indicate that there is a need to intensify research in this field or form international alliances against these parasites [62]. In this sense, the flexibility and potential of MEVs puts them at the forefront of modern tools for delivering cross-protective determinants for parasitic disease control, in line with the One Health approach.

## Figures and Tables

**Table 2:** Helminth parasite (*Ascaris suum*, *Brugia malayi*, *Echinococcus granulosus*, *E. multilocularis*, *Fasciola hepatica*, *Schistosoma japonicum*, *S. mansoni*, *Taenia solium*, *T. crassiceps*, and *Trichinella spiralis*), multiepitope vaccine, adjuvant system; administration dose, route and schedule of vaccination and booster, challenge, protection percentage in terms of parasite burden recovery, survival after a lethal challenge or lesions induced by the challenge in comparison to adjuvant control animals, humoral and cellular immune response studied, and authors of the studies included.

Parasite	Antigen	Adjuvant	Dose/Route/Schedule	Challenge/Administration Route	Vaccine Efficacy (% Reduction)	Humoral Response	Cellular Response	Authors, Publication Year [Reference]
Trematodes								
*F. hepatica*	B6, T14 (B, T epitopes)	ADAD	10 μg s.c./3 doses/2 w interval	7 metacercariae/v.o.	31% hepatic lesion, 57.1% survival	No study	No study	Rojas-Caraballo et al., 2017 [16]
*F. hepatica*	B1, B5, B6 (B epitopes)	ADAD	10 μg s.c./3 doses/2 w interval	7 metacercariae/v.o.	14% hepatic lesion, 57.1% survival	No study	No study	Rojas-Caraballo et al., 2017 [16]
*F. hepatica*	T14, T15, T16 (T epitopes)	ADAD	10 μg s.c./3 doses/2 w interval	7 metacercariae/v.o.	45% hepatic lesion, 71.4% survival	↑IgG, IgG1	↑IL-12, IL-10, IL-8	Rojas-Caraballo et al., 2017 [16]
*F. hepatica*	B1, B2, B5, B6, T14, T15, T16 (B, T epitopes)	ADAD	10 μg s.c./3 doses/2 w interval	7 metacercariae/v.o.	39% hepatic lesion, 57.1% survival	No study	No study	Rojas-Caraballo et al., 2017 [16]
*S. japonicum*	SjPGAM-SjEnol	Montanide ISA 206	27 μg s.c./3 doses/2 w interval	40 cercariae/i.p.	39.7% adult worm, 64.9% liver egg	↑IgG	No study	Guo et al., 2010 [25]
*S. mansoni*	Pmy-3, TPI-1, TPI-2, Sm23, Sm28-1, Sm28-2, Sm28-3, Smcal (Polymer-1); Pmy-1, Pmy-2, TPI-1, TPI-2, Sm23, Sm28-1, Sm28-2, Sm28-3 (Polymer-2) (B, T epitopes)	Freund’s adjuvant	50 μg i.m./3 doses/2 w interval	120 cercariae/p.c.	No protection	↑IgG	No study	Yang et al., 2000 [24]
*S. mansoni*	Pmy-1, Pmy-2, TPI-1, TPI-2, Sm23, Sm28-1, Sm28-2, Sm28-3 (B, T epitopes)	No adjuvant	100 μg i.m./3 doses/3 w interval	150 cercariae/p.c.	No protection	No study	No study	Yang et al., 2000 [24]
Cestodes								
*E. granulosus*	Eg95, Eg14-3-3, EgEnolase (B, T epitopes)	Freund’s adjuvant	0.5 mg/mL s.c./2 doses/4 w interval	105,000 protoscoleces/v.o.	100% intestine adults	↑IgG, IgE	↑IL-4	Pourseif et al., 2021 [20]
*E. granulosus*	Eg95, Eg14-3-3, EgEnolase (B, T epitopes)	Freund’s adjuvant	1 mg/mL s.c./2 doses/4 w interval	2000 eggs/v.o.	85.4% liver cysts	↑IgG, IgE	↑IL-4	Pourseif et al., 2021 [20]
*E. granulosus*	EgA31, EgTrp, EgGST, Eg95, P14-3-5 (T epitopes)	Freund’s adjuvant	50 μg s.c./3 doses/2 w interval	500 protoscoleces/i.p.	99.6% peritoneal cysts	No study	No study	Esmaelizad et al., 2013 [21]
*E. multilocularis*	EMY162, LAP, GLUT1 (B, T epitopes)	Freund’s adjuvant	50 μg i.p./4 doses/1 w interval	1000 protoscoleces/i.p.	50.0% liver cysts, 96.9% cysts weight	↑IgG	↑IFN-γ, IL-4, CD4+, CD8+	Zhou et al., 2023 [22]
*T. crassiceps*	KETc1, KETc12, GK1, KETc7	No adjuvant	5 × 10^10^ phage s.c./4 doses/0, 12, 23, 34 d	10 cysticerci/i.p.	65.9% peritoneal cysticerci	No study	No study	Manoutcharian et al., 2004 [23]
*T. solium*	KETc1, KETc12, GK1, KETc7	No adjuvant	4 × 10^11^–4 × 10^12^ phage s.c./2 doses/2 w interval	17,000 eggs/v.o.	95.1% muscle cysticerci	↑IgG	↑IFN-γ, IL-4. Lymphocyte proliferation	Manoutcharian et al., 2004 [23]
*T. solium*	KETc1, KETc12, GK1, KETc7	No adjuvant	4 × 10^12^ phage v.o./2 doses/2 w interval	17,000 eggs/v.o.	41.7% muscle cysticerci	= IgG	↑IFN-γ, IL-4. Lymphocyte proliferation	Manoutcharian et al., 2004 [23]
Nematodes								
*A. suum*	Top 35 immunogenic (B epitopes)	BpMPLA	25 μg s.c./2 doses/10 d interval	2500 embryonated eggs/v.o.	33.7% lung larvae	↑IgG1, IgG3, IgE. Low IgG2a	↑IL-4, IL-5, IL-13 in lung, ↑ lymphocyte, eosinophil.↓ neutrophils	Gazzinelli-Guimaraes et al., 2022 [26]
*B. malayi*	TRX, TGA, ALT-2 (B, T epitopes)	Alum	50 μg i.p./4 doses/2 w interval	20 L3 larvae/i.p.	69.5% implanted larvae	↑IgG, IgG1, IgG2a, IgG2b	↑IL-5, IFN-γ. = IL-4, IL-2, IL-10. Splenocyte proliferation	Anugraha et al., 2015 [12]
*B. malayi*	1–30 (EDI), 89–128 (EDII) of ALT-2 (B epitopes)	Alum	50 μg i.p./4 doses/1 w interval	10 L3 larvae i.p.	74.6% implanted larvae	↑IgG1, IgG2b. = in IgGa, IgG3, IgA, IgM	No study	Madhumathi et al., 2017 [13]
*B. malayi*	TRXP1, TRXP2 (B, T epitopes)	Alum	100 μg i.p./4 doses/1 w interval	10 L3 larvae i.p.	75.1% implanted larvae	No study	↑IL-2 IL-5. = IL-4, IL-10 and IFN-γ	Madhumathi et al., 2010 [14]
*B. malayi*	TGA, TRX P1, TRX P2 (B, T epitopes)	Alum	100 μg i.p./4 doses/1 w interval	10 L3 larvae/i.p.	63.0% implanted larvae	↑IgG1, IgM	↑IL-2, IFN-γ, IL-4, IL-5, IL-10. Splenocyte proliferation	Immanuel et al., 2017 [15]
*T. spiralis*	T2, T5 (T epitopes), YX1 (B epitope)	Freund’s adjuvant	30 μg s.c./3 doses, 2 w interval	400 muscle larvae/v.o.	35.5% muscle larvae	↑IgG, IgG1, IgG2a	↑IFN-γ, IL-2, IL-4, IL-5, IL-6, Tfh, GC.↓ Tfr, Treg.	Gu et al., 2020 [17]
*T. spiralis*	YX1 (B epitope)	Freund’s adjuvant	30 μg s.c./3 doses/2 w interval	400 muscle larvae/v.o.	12.4% muscle larvae	↑IgG1 ↓ IgG2a	= cytokines.↑ Tfh, GC.↓ Tfr, Treg	Gu et al., 2020 [17]
*T. spiralis*	YX1-KLH, 8F7-KLH, M7-KLH epitopes	Montanide ISA 50 V2	50 μg s.c./3 doses/2 w interval	400 muscle larvae/v.o.	35.0% muscle larvae	↑IgG, IgG1	No study	Gu et al., 2013 [18]
*T. spiralis*	P2, P3, P4, P5 (T epitopes), YX1 (B epitope)	Montanide ISA 50 V2	25 μg s.c./3 doses/2 w interval	400 muscle larvae/v.o.	55.4% muscle larvae	↑IgG, IgG1, IgG2	↑IFN-γ, IL-4, IL-5	Gu et al., 2017 [19]

TRX: thioredoxin; TGA: transglutaminase; ALT-2: abundant larval transcript-2; EDI: epitope domain I; EDII: epitope domain II; TRXP1: thioredoxin epitopic region 1 (2–27); TRXP2: thioredoxin epitopic region 2 (94–107/109); LAP: leucine aminopeptidase; Pmy-1: paramyosin (1–25); Pmy-2: paramyosin (481–500); Pmy-3: paramyosin (355–367); TPI-1: triose phosphate isomerase (142–154); TPI-2: triose phosphate isomerase (194–210); Sm23: integral membrane protein (143–163); Sm28-1: glutathione transferase (10–43); Sm28-2: glutathione transferase (115–131); Sm28-3: glutathione transferase (190–211); Smcal: calpain (344–353); Tfh: T follicular helper cells; GC: germinal center B cells; YX1: protective B epitope of *T. spiralis* paramyosin (Ts-Pmy); YX1-KLH: YX1 conjugated with keyhole limpet hemocyanin (KLH); 8F7-KLH: Ts87 conjugated with keyhole limpet hemocyanin (KLH); M7-KLH: M7 conjugated with keyhole limpet hemocyanin (KLH); BpMPLA: monophosphoryl lipid A *B. pertussis*. Time in weeks (w) or days (d). Administration route: subcutaneous (s.c.), oral (v.o.), intraperitoneal (i.p.), intramuscular (i.m.), or percutaneous (p.c.). Changes in humoral and cellular immune response: no changes (=), increase (↑), and decrease (↓).

## Data Availability

Data are provided within the manuscript.

## Supplementary Materials

The following supporting information can be downloaded at https://www.mdpi.com/article/10.3390/biom15060867/s1. Table S1: Search strategy.
