# Supplementary material for: Current Efficacy of Multiepitope Vaccines Against Helminths: A Systematic Review
Source: Biomolecules. 2025 Jun 13;15(6):867. doi: 10.3390/biom15060867 (PMC12190703; doi:10.3390/biom15060867)
Supplement: Supplementary file 1 [file biomolecules-15-00867-s001.zip › biomolecules-3553348-supplementary.pdf]

Supplementary Table S1. Search strategy.

Search strategy				
**Concept 1**		**Concept 2**		**Concept 3**
vaccin*	**AND**	multiepitop* **OR** multi-epitop* **OR** multivalent* **OR** multi-valent*	**AND**	fasciol*
				schistosom*
				clonorch*
				paragonim*
				heteroph*
				opisthorch*
				metagonim*
				fasciolop*
				echinococ* **OR** hydatid*
				taen* **OR** cysticerc*
				diphyllobot*
				enterobi* **OR** pinwor*
				brugi* **OR** filari*
				ascari*
				trichine*
				haemonch*
				wuchereri*
				trichuri* **OR** whipwor*
				ancylostom* **OR** hookwor*
				necato* **OR** hookwor*
				strongyloid*
				anisaki*
				dracuncul*

Search equation

Web of Science:

vaccin* (Topic) AND multiepitop* OR multi-epitop* OR multivalent* OR multi-valent* (Topic) AND fasciol* OR schistosom* OR clonorch* OR paragonim* OR heteroph* OR opisthorch* OR metagonim* OR fasciolop* OR echinococ* OR hydatid* OR taen* OR cysticerc* OR diphyllobot* OR enterobi* OR pinwor* OR brugi* OR filari* OR ascari* OR trichine* OR haemonch* OR wuchereri* OR trichuri* OR whipwor* OR ancylostom* OR hookwor* OR necato* OR hookwor* OR strongyloid* OR anisaki* OR dracuncul* (Topic)

PubMed:

"vaccin*"[All Fields] AND ("multiepitop*"[All Fields] OR "multi epitop*"[All Fields] OR "multivalent*"[All Fields] OR "multi valent*"[All Fields]) AND ("fasciol*"[All Fields] OR "schistosom*"[All Fields] OR "clonorch*"[All Fields] OR "paragonim*"[All Fields] OR "heteroph*"[All Fields] OR "opisthorch*"[All Fields] OR "metagonim*"[All Fields] OR "fasciolop*"[All Fields] OR "echinococ*"[All Fields] OR "hydatid*"[All Fields] OR "taen*"[All Fields] OR "cysticerc*"[All Fields] OR "diphyllobot*"[All Fields] OR "enterobi*"[All Fields] OR "pinwor*"[All Fields] OR "brugi*"[All Fields] OR "filari*"[All Fields] OR "ascari*"[All Fields] OR "trichine*"[All Fields] OR "haemonch*"[All Fields] OR "wuchereri*"[All Fields] OR "trichuri*"[All Fields] OR "whipwor*"[All Fields] OR "ancylostom*"[All Fields] OR "hookwor*"[All Fields] OR "necato*"[All Fields] OR "hookwor*"[All Fields] OR "strongyloid*"[All Fields] OR "anisaki*"[All Fields] OR "dracuncul*"[All Fields])
